# Assessment of hand hygiene adherence using a web camera

**DOI:** 10.1186/1753-6561-5-S6-P104

**Published:** 2011-06-29

**Authors:** H Kunishima, K Tokuda, M Meguro, T Kobayashi, J Chiba, T Aoyagi, M Hatta, M Kitagawa, Y Honda, M Kaku

**Affiliations:** 1 Infection Control and Laboratory Diagnostics, Tohuku University Graduate School, Sendai-city, Japan; 2 Infection Control Team, Sendai Kosei Hospital, Sendai-city, Japan

## Introduction / objectives

The most effective method of controlling nosocomial infection is hand hygiene. Hand hygiene has been monitored by trained nurses’ observation of hygiene practices and assessment of the amount of alcohol-based hand sanitizer used. This study used a web camera to examine hand hygiene adherence.

## Methods

A web camera was installed in the ICU in September 2010, video from the camera was relayed to the Department of Infection Control via the hospital’s intranet, and that video was saved on a server for later observation. Starting in December 2010, personnel on both the day and night shifts were assessed for a total of 100 hours using the Observation Form of the WHO Patient Safety team.

## Results

Alcohol-based hand sanitizer were used 170L per 1,000 patients. Conventional observation by ward nurses indicated that compliance with hand hygiene before touching a patient was 77%. Direct observation with the web camera indicated that hand hygiene was required 11.6 times/hour for each patient. Adherence to hand hygiene was 22.5%. Compliance with hand hygiene was 25.3? before touching a patient, 25.2? before a clean/aseptic procedure, 14.0? after body fluid exposure risk, 30.6? after touching a patient, and 11.5? after touching patient surroundings. After improved education of and practices by health care practitioners, staff were again observed for 100 hours. Adherence to hand hygiene was found to have improved to 33.8% (P < 0.05).

## Conclusion

Direct observation with a web camera allowed video to be recorded and saved for long periods and it allowed practices to be assessed. The saved video was circulated among the health care practitioners to allow a more objective intervention.

## Disclosure of interest

None declared.

